# Formation of reflexive skills as prevention of suicidal behavior in adolescents

**DOI:** 10.1192/j.eurpsy.2022.2176

**Published:** 2022-09-01

**Authors:** N. Galiautdinova, S. Galyautdinova, L. Galyautdinova

**Affiliations:** 1 Bashkir State University, Department Of Psychology, Ufa, Russian Federation; 2 Moscow State Psychological Pedagogical University, Faculty Of Extreme Psychology, Moscow, Russian Federation; 3 Republican Clinical Psychiatric Hospital, Child Psychiatry, Ufa, Russian Federation

**Keywords:** reflection, Suicide, teenagers

## Abstract

**Introduction:**

An important task of psychiatrists and psychologists is the prevention of suicidal behavior in adolescents.

**Objectives:**

Highlighting the stages of the formation of reflexive skills for the development of training sessions on the prevention of adolescent suicide.

**Methods:**

Analysis of the results of theoretical and empirical studies of reflection and suicide by psychiatrists and psychologists.

**Results:**

Reflexive skills are a system of deliberate actions aimed at understanding and evaluating “I” and one’s own behavior. Theoretical analysis made it possible to distinguish three stages in the formation of reflexive skills. EMOTIONAL - evaluation of “I” as the basis of positive emotions. COGNITIVE - understanding (awareness) of one’s own capabilities for solving a problem. OPERATIONAL - solving a problem based on an assessment of the “I” and an understanding of their capabilities.

**Conclusions:**

The identification of three stages of the formation of reflexive skills, as prevention of adolescent suicide, makes it possible to develop an effective training program for adolescents at risk in the Centers for psychiatric and psychological assistance.

**Disclosure:**

No significant relationships.

